# Value of machine learning-based transrectal multimodal ultrasound combined with PSA-related indicators in the diagnosis of clinically significant prostate cancer

**DOI:** 10.3389/fendo.2023.1137322

**Published:** 2023-03-08

**Authors:** Maoliang Zhang, Yuanzhen Liu, Jincao Yao, Kai Wang, Jing Tu, Zhengbiao Hu, Yun Jin, Yue Du, Xingbo Sun, Liyu Chen, Zhengping Wang

**Affiliations:** ^1^ Department of Ultrasound, The Affiliated Dongyang Hospital of Wenzhou Medical University, Dongyang, China; ^2^ Department of Ultrasound, Cancer Hospital of the University of Chinese Academy of Sciences, Zhejiang Cancer Hospital, Hangzhou, China; ^3^ Institute of Basic Medicine and Cancer (IBMC), Chinese Academy of Sciences, Hangzhou, China; ^4^ Key Laboratory of Head & Neck Cancer Translational Research of Zhejiang Province, Hangzhou, Zhejiang, China

**Keywords:** clinically significant prostate cancer, multimodal ultrasound, serum prostate specific antigen, machine learning, artificial neural network

## Abstract

**Objective:**

To investigate the effect of transrectal multimodal ultrasound combined with serum prostate-specific antigen (PSA)-related indicators and machine learning for the diagnosis of clinically significant prostate cancer.

**Methods:**

Based on Gleason score of postoperative pathological results, the subjects were divided into clinically significant prostate cancer groups(GS>6)and non-clinically significant prostate cancer groups(GS ≤ 6). The independent risk factors were obtained by univariate logistic analysis. Artificial neural network (ANN), logistic regression (LR), support vector machine (SVM), decision tree (DT), random forest (RF), and K-nearest neighbor (KNN) machine learning models were combined with clinically significant prostate cancer risk factors to establish the machine learning model, calculate the model evaluation indicators, construct the receiver operating characteristic curve (ROC), and calculate the area under the curve (AUC).

**Results:**

Independent risk factor items (P< 0.05) were entered into the machine learning model. A comparison of the evaluation indicators of the model and the area under the ROC curve showed the ANN model to be best at predicting clinically significant prostate cancer, with a sensitivity of 80%, specificity of 88.6%, F1 score of 0.897, and the AUC was 0.855.

**Conclusion:**

Establishing a machine learning model by rectal multimodal ultrasound and combining it with PSA-related indicators has definite application value in predicting clinically significant prostate cancer.

## 1 Introduction

Prostate cancer (PCa) is the second most common malignant tumor in men worldwide and the fifth leading cause of death globally (1, 2). In recent years, with changes in lifestyle and the acceleration of aging, the incidence of PCa in the world also has increased year by year (1, 3). Zattoni classified prostate cancer into clinically significant prostate cancer (CS-PCa) according to its Gleason score (GS) and tumor invasion range, and they compared it to non-clinically significant PCa (Non-CS-PCa) (4). Non-CS-PCa has low aggressiveness and slow progression, and more follow-up and active monitoring are used without intervention treatment (5). CS-PCa is highly invasive and progresses rapidly. If not actively treated, it can lead to systemic metastasis, causing serious harm to patients’ lives and quality of life (6). Data show that at the initial diagnosis, more than half of PCa patients already have bone metastases, accompanied by bone pain, pathological fractures, movement disorders, spinal cord compression, and other complications (7). Hormonal therapy, radiotherapy, chemotherapy, and surgical treatment should be chosen according to the stage of the illness (8). The incidence of CS-PCa is higher in older men and men with no family history (9). Early detection of CS-PCa is of profound significance for the effective treatment of patients.

The difficulty with diagnosing and treating prostate cancer lies in the different biological behaviors of the tumors, and the characteristics of prostate cancer are multifocal, microfocal, and heterogeneous (10). Currently, a systematic ultrasound-guided biopsy is the main method for diagnosing prostate cancer. However, it can miss CS-PCa and overdiagnose Non-CS-PCa (11–13).

The clinical value of prostate-specific antigen (PSA) as a marker of prostate tumor has been recognized, but the high false positive rate for PSA can make it difficult to accurately diagnose PCa (14). Currently, researchers are exploring non-invasive imaging techniques to increase the accuracy of PCa diagnosis. In auxiliary imaging examinations, multi-parameter NMR has shown obvious advantages (15). Multimodal ultrasonography (MUS) refers to the combined application of transmittal color ultrasound (TRUS), transrectal real-time elastography (TRTE), and transrectal contrast enhanced ultrasound (TR-CEUS) technology to perform diagnostic rectal contrast enhanced ultrasound on diseases. Compared to using any of these technologies alone, a combined examination has been shown to improve the diagnosis rate (16). As the availability of transmittal MUS and multiparameter magnetic resonance in prostate cancer detection continues to improve, more studies are reporting the practicability of combining serum biomarkers with imaging evaluation. In order to improve the specificity of CS-PCa detection and avoid over-diagnosis of non-CS-PCA, a CS-PCa machine learning model was established and predicted by combining MUS and PSA-related risk indicators.

## 2 Materials and methods

### 2.1 Sources of the data

**Figure 1:**
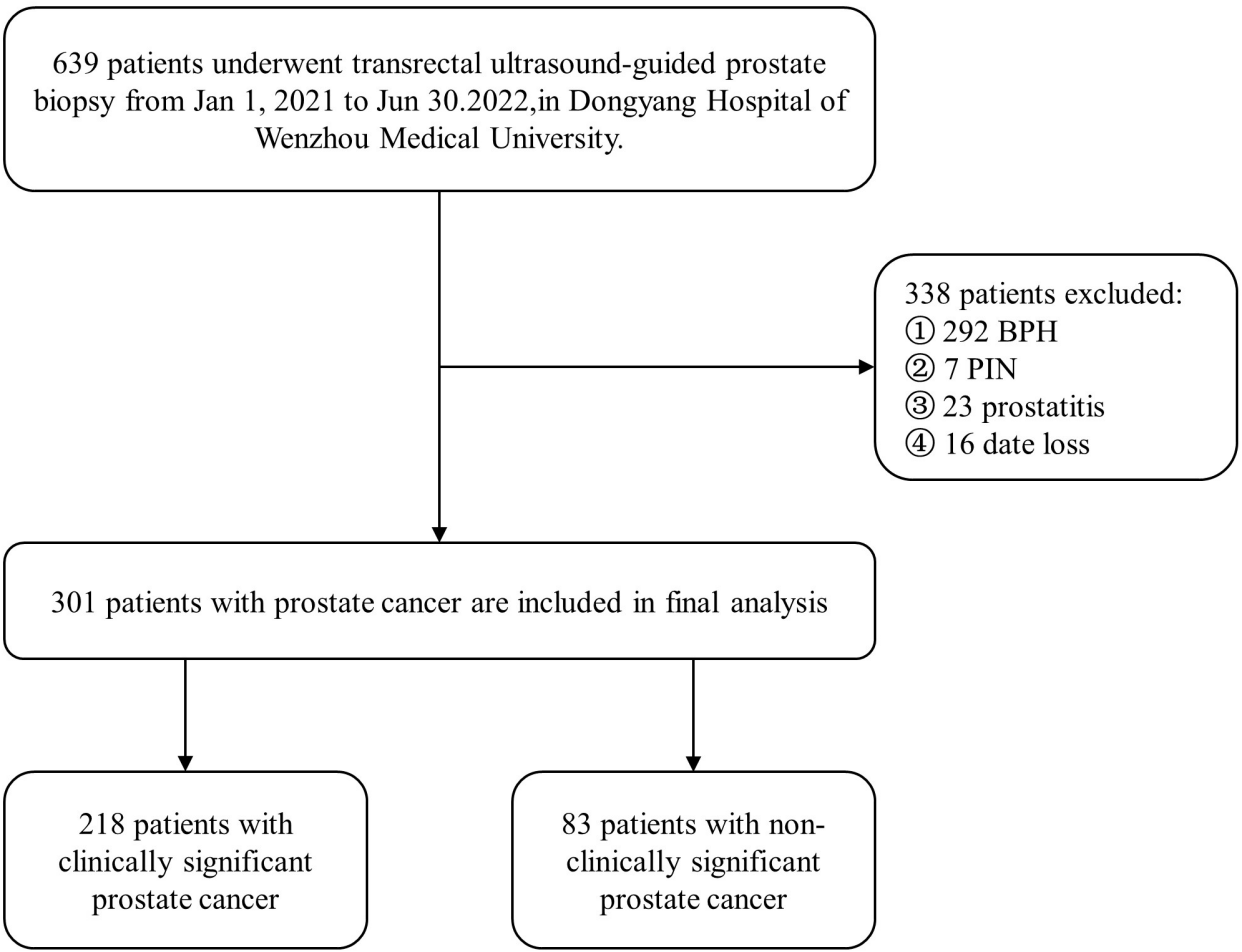
Flow chart of inclusion and exclusion of the study population.

A total of 639 clinically suspected patients with prostate cancer at Dongyang People’s Hospital from January 2021 to June 2022 were retrospectively analyzed. Relevant examinations and transmittal MUS were performed before surgery, and the pathological results of the prostate biopsy operation were taken as the “gold standard.” Of the 301 eligible prostate cancer patients (according to the pathological results of GS), 218 were placed in the CS-PCa group, and 83 were placed in the Non-CS-PCa group. Their average age was 75 ± 7.35 years, with a range from 53 to 90 years old. The samples were screened according to the following inclusion criteria (1): the patients underwent prostate biopsy or radical resection for prostate cancer, and PCa was pathologically confirmed, including GS (2). Serum PSA-related indexes, namely serum total PSA (tPSA), free serum prostate-specific antigen (fPSA), and the f/tPSA ratio were determined before operation (3). A preoperative TRUS examination, TRTE examination, and CEUS examination were performed (4). Each patient had complete clinical data. The exclusion criteria were these (1): The patient had received other prostate treatments before surgery, such as endocrine therapy and radiation therapy (2). Postoperative pathology suggested pathologic types other than PCa (3). Patients did not have complete clinical data and an imaging examination. **Figure 1** is a flow chart of the selection process.

The study was performed in accordance with the ethical guidelines of the Helsinki Declaration. It was approved by The Affiliated Dongyang Hospital of Wenzhou Medical University(IRB: 2022-YX-306). Due to the retrospective nature of the study and the use of anonymized patient data, written informed consent for participation was waived.

### 2.2 Instruments and methods

#### 2.2.1 Instruments

PSA-related indexes were determined by the Abbott automatic immunochemical analyzer, and the Abbott PSA reagent was used. The Saute CLASS C ultrasonic diagnostic instrument of Parson Company in Italy was used with a double-plane rectal probe with a frequency of 3-13 MHz. SonoVue (Bracco, 59mg/piece) was used as the contrast agent.

#### 2.2.2 PSA series of indicators determination method

The tPSA, fPSA, and the f/tPSA ratio were determined by immunochemiluminescence. For the TRUS test and scoring method, the patient was placed in the left lateral position, and the TRUS test was performed. Left and right diameters (W), enterobacteria diameters (H), and upper and lower diameters (L) were obtained on standard prostate sections. Prostate volume (PV) was calculated according to Equation 1, and prostate volume (PV) was prostrate.


PV= 0.523*L*W*H

**Figure 2:**
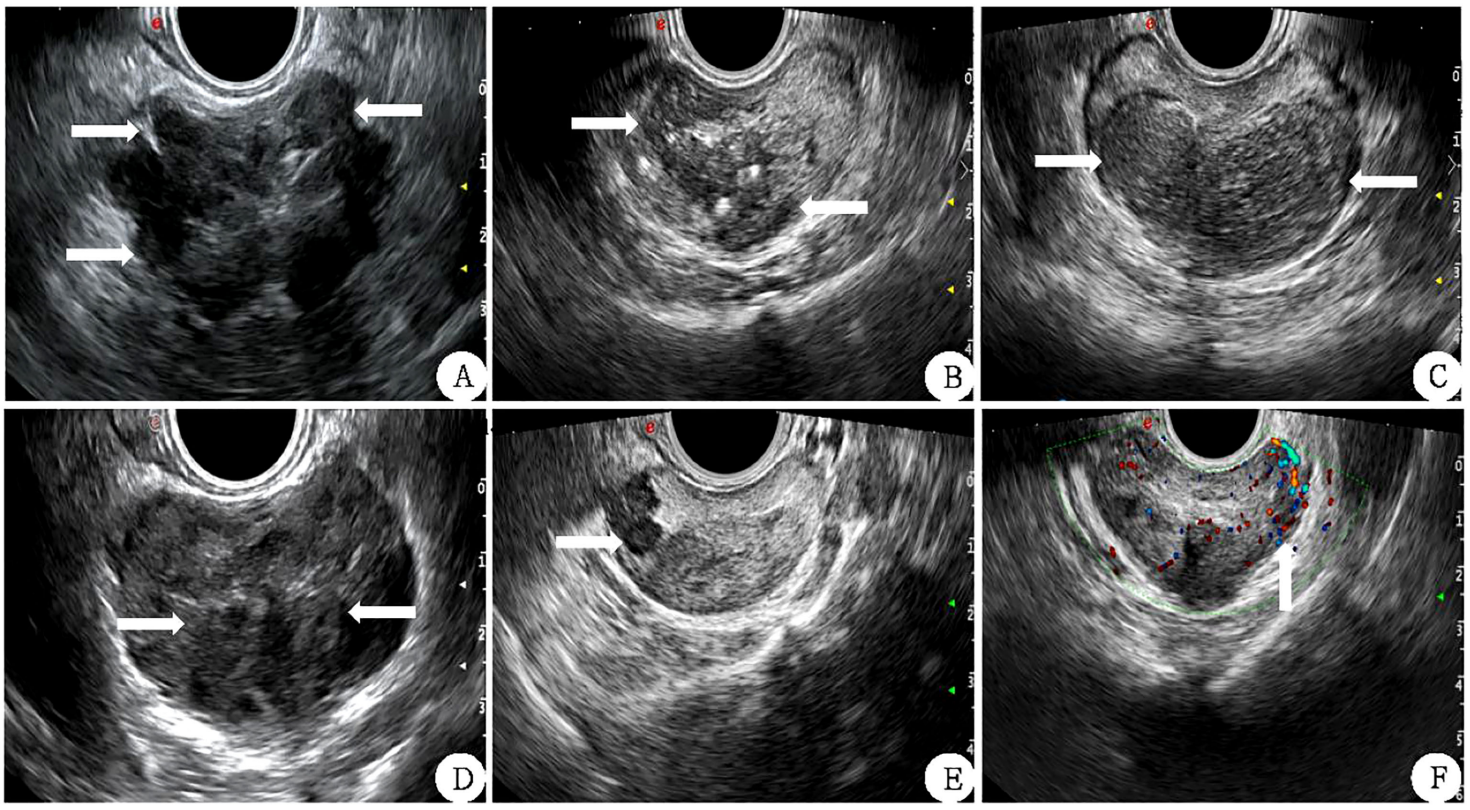
Correlation features of prostate 2D ultrasound image scores (white arrow) **(A)** The lateral lobe capsule is not smooth and protrudes outwards. **(B)**: the echo of the inner and outer glands is uniform and the boundary is not clear. **(C)** left and right lobes are not symmetrical. **(D)** The boundary between the inner and outer glands is unclear. **(E)** Hypoechoic nodules can be seen in the right external gland. **(F)** There is abundant blood flow in the left external gland nodule.

Inner gland PV (IGPV) was calculated according to the above method, and external gland PV (EGPV) was calculated as the difference between PV and IGPV. Prostate specific antigen density (PSAD) and inner gland specific antigen density (Inner Gland) were obtained by applying the ratio of tPSA to PV, IGPV, and EGPV PSAD, IGPSAD), external gland PSAD (EGPSAD) density. The score of two-dimensional ultrasound (2D-US) used the correlation feature standard of two-dimensional prostate images (17, 18) (1): Bilateral lobe asymmetry of prostate (2); Uneven echo of prostatic parenchyma (3); Incomplete prostate capsule (4); The boundary between the inner and outer prostate glands was not clear (5); There were hypothetic nodules or diffuse gland lesions in the prostate (6); There was blood flow in hypothetic nodules or in diffuse glandular lesions, as shown in **Figure 2**. Each of the above six features that meet the requirements is considered as 1 point, which will be added up one by one. The lowest score is 0 points (none set), and the highest score is 6 points (all men).

#### 2.2.3 TRTE inspection and scoring method

**Figure 3:**
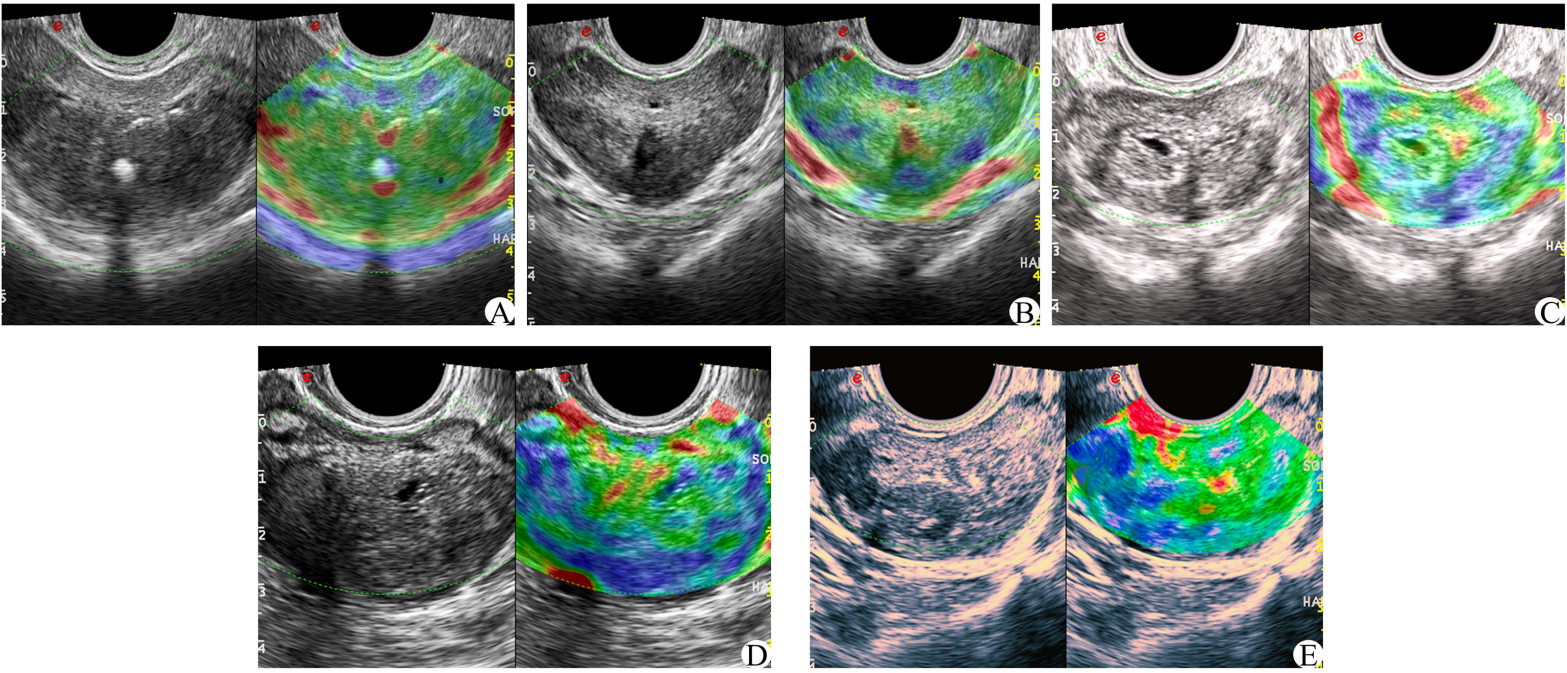
Zordo 5-point evaluation sample; **(A)** The whole prostate showed uniform green color at 1 point; **(B)** The whole gland is symmetrically distributed in blue and green phases. **(C)** 3 parts in the prostate with asymmetric distribution of texture hardening area; **(D)** 4 points of hypothetic nodules in the prostate, with hard central texture and soft peripheral texture; **(E)** Hypoechoic nodules in the prostate, and the whole nodules become hard in texture.

In the elastic imaging mode, the maximum cross-section of a lesion was chosen for the elastic score. If no suspicious lesion was noted by conventional ultrasound, the maximum cross-section of the prostate was selected. The physician used the probe to manually press the prostate with regular frequency to determine the elastic score for the suspected lesion and judge whether it was benign or malignant. Scoring was based on the 5-point method proposed by Zordo (19). There were five possible outcomes, as shown in **Figure 3** (1): Benign: there is a uniform strain of the whole gland (receiving 1 point); it appeared uniformly green (2); Probably benign, the whole gland is symmetrical but not uniform strain, uneven blue and green with 2 points (3); Uncertain, no clear lesions were found in the whole gland, and the blue elastic map was 3 points (4); It may be malignant; the edge of the lesion is green, and the center is blue (5); Indicates malignancy, there is no strain in and around the lesion, and the blue color is 5 points.

#### 2.2.4 Transrectal CEUS examination and scoring method

**Figure 4:**
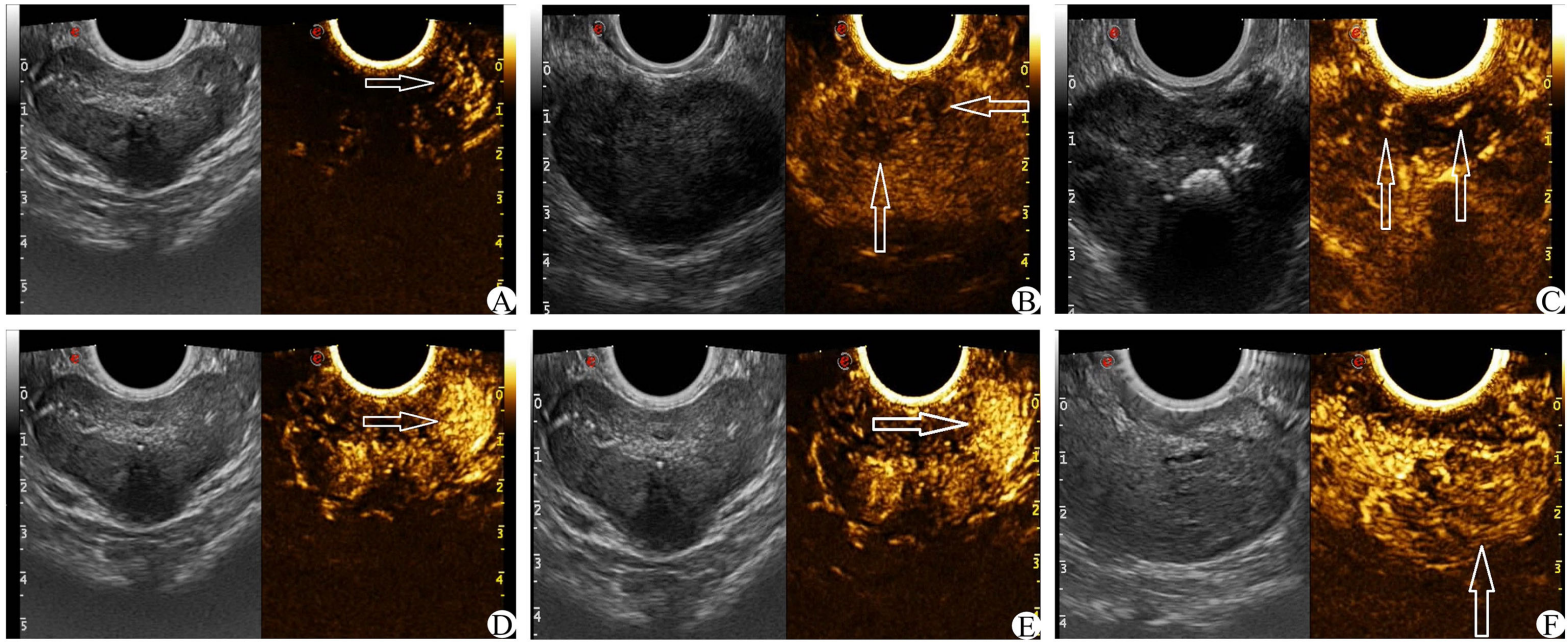
CEUS score (white arrow) **(A)**: The arrival time of contrast media in the left lesion was earlier than that in the contralateral side. **(B)** The enhancement was uneven and the boundary was not clear. **(C)** Enhance the asymmetric vascular structure in the lesion. **(D)** The peak intensity of the left lesion was higher than that of the contralateral lesion. **(E)** The enhanced boundary of the left lesion was clear. **(F)** The clearance time of the contrast agent in the lesion was shorter than that of surrounding normal tissue.

On the basis of conventional TRUS, the suspect target was locked and determined. The contrast agent was 5 ml of normal saline. After full oscillation, 4.8 ml dilutions of the contrast agent were extracted and injected through the patient’s cubital vein mass. Then, 5 ml normal saline was injected rapidly. The CEUS was observed continuously for 2 min. The suspicious target area was focused on the fixed section in the original 40 s, and the images were dynamically stored. After 40 s, the entire prostate was scanned completely, and the dynamic image storage was continued until the end of the 2 min and stopped. The CEUS score was obtained by observing the relevant features of prostatic angiography images (17, 20, 21) (1): The arrival time of the lesions was earlier than that of the surrounding prostate tissue (2); The peak intensity of the lesion was higher than that of the surrounding prostate tissue (3); Asymmetric vascular structure appeared at the beginning of lesion enhancement (4); The enhancement boundary was clear after the enhancement (5); There was uneven enhancement of the intravenous contrast agent (6); The clearance time of the contrast agent in the lesion was shorter than that of surrounding prostate tissue (see **Figure 4**). Each of the six features above that met the requirements was considered as 1 point, which were later summed. The lowest score was 0 points (none set), and the highest score was 6 points (all men).

#### 2.2.5 Pathological GS classification group and the CS-PCa and Non-CS-PCa groups

PCa was divided into 5 grades according to the 2021 edition of “Consensus on Standardized Specimen Sampling and Pathological Diagnosis of Prostate Cancer” (22). PCa was separated into CS-PCa and Non-CS-PCa according to the Gleason score and the tumor invasion range, and the diagnostic criteria for CS-PCa were Epstein criteria (23). In this study, if the postoperative GS of each patient had multiple scores, the one with the highest score was selected.

The ultrasound image score and pathological results of GS were scored by two physicians with more than 8 years of experience in prostate ultrasound work. If the scoring results were consistent, they were adopted; if there were differences, a third physician with more than 10 years of relevant experience made the judgment.

### 2.3 Statistical analysis and modeling

SPSS statistical software was used for data collation and analysis. Unary logistic regression was used to analyze the independent risk factors of significant prostate cancer, and the independent risk factor group was used to establish the machine learning model. Python 3.8 programming language combined with the Scikit-learn 1.1 machine learning library was used to establish the machine learning prediction model of CS-PCa, and the ROC curve was established to calculate the AUC. Sensitivity, specificity, positive predictive value(PPV), negative predictive value(NPV), F1 score, and Youden index were combined to evaluate the efficacy of the model for CS-PCa, and the Kappa coefficient was used to measure the agreement between the model classification and the actual results.

## 3 Results

### 3.1 Analysis of variables associated with clinically significant prostate cancer

The dependent variable was whether the outcome was CS-PCa (yes = 1, no = 0), and age, tPSA, fPSA, fPSA/tPSA, PV, IGPV, EGPV, PSAD, IGPSAD, EGPSAD, 2D-US score, CEUS score, and elasticity score were the independent variables for univariate logistic regression analysis. The results showed in **Table 1** that the greater the age, the higher the tPSA, fPSA, PSAD, IGPSAD, EGPSAD, 2D-US score, CEUS score, and elasticity score and the lower the fPSA/tPSA. The 10 factors were independent risk factors for predicting clinically significant prostate cancer (P< 0.05) as an input feature for building the machine learning model.

**Table 1:** Logistic regression analysis of variables related to clinically significant prostate cancer.

Variable	Univariate Logistic regression analysis	
	OR*(95%CI*)	*P*
Age	1.044 (1.008, 1.081)	0.017
tPSA	1.582 (1.392, 1.797)	<0.001
fPSA	1.516 (1.210, 1.900)	<0.001
fPSA/tPSA	<0.001(<0.001,0.001)	<0.001
PV	0.995 (0.984, 1.007)	0.429
IGPV	0.996 (0.981, 1.012)	0.663
EGPV	0.983 (0.954, 1.013)	0.260
PSAD	7432.64 (507.164, 108927.445)	<0.001
IGPSAD	86.907 (21.514, 351.065)	<0.001
EGPSAD	1.981 (1.388, 2.828)	<0.001
2D-US	2.931 (2.130, 4.034)	<0.001
CEUS	2.445 (1.963, 3.044)	<0.001
TRTE	2.607 (1.891, 3.594)	<0.001

*OR, odds ratio; CI, confidence interval.

### 3.2 Machine learning model analysis of clinically significant prostate cancer

**Figure 5:**
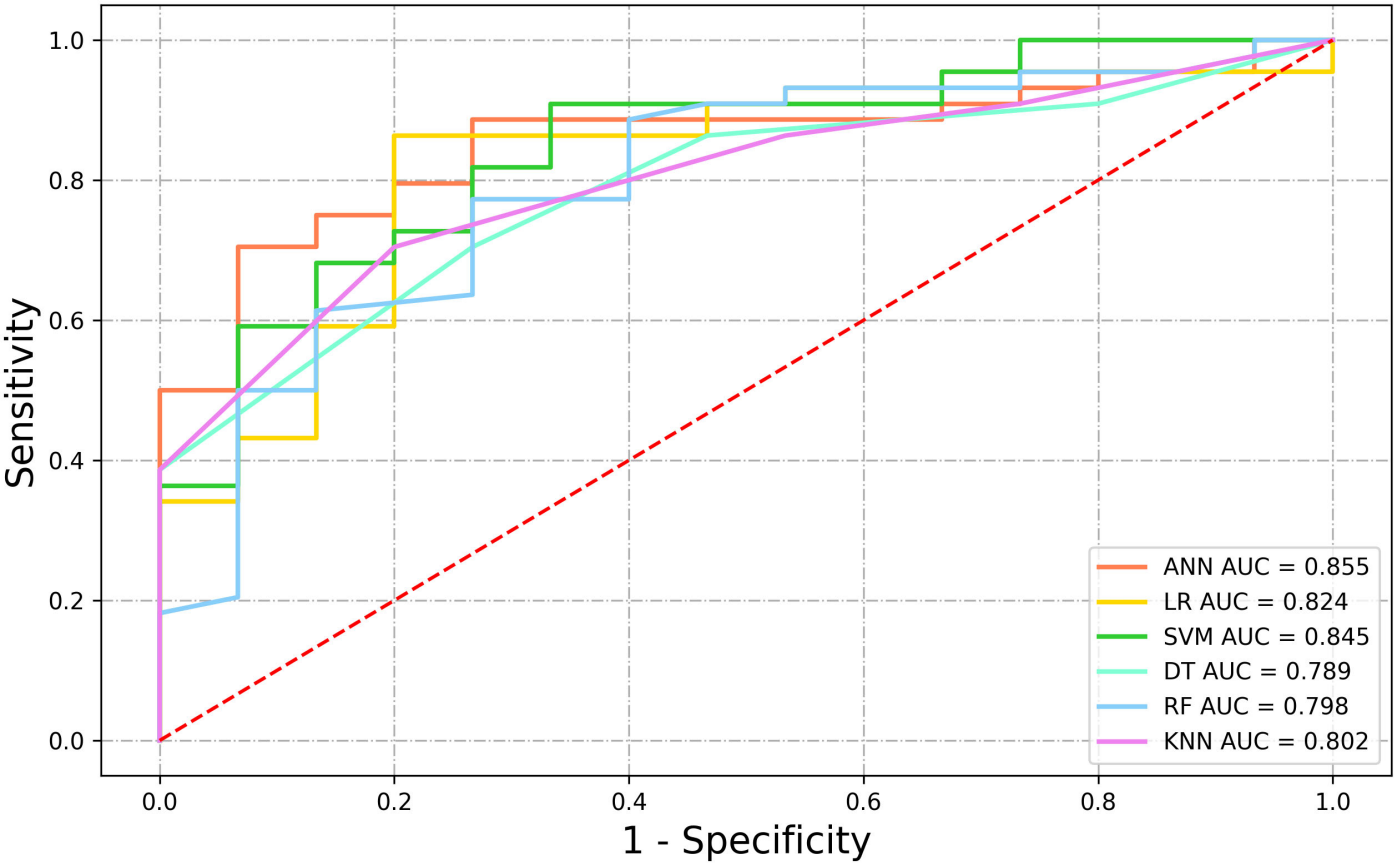
ROC curves of six machine learning models.

The data was divided into training set and test set according to the ratio of 8:2. There were 240 patients in the training set with an average age of 74 ± 7.90, and 61 patients in the test set with an average age of 75 ± 7.18. This data is preprocessed by two methods. Continuous variables such as age, tPSA, fPSA, fPSA/tPSA, PSAD, IGPSAD and EGPSAD feature variables have different dimensions and magnitude. Data normalization is used to eliminate the dimension effect and scale different data to the same magnitude. Discrete variables such as 2D-US, CEUS and TRTE features, due to the factors of different categories of discrete value data, one-hot encoding is used to process this type of data, which also plays a role in expanding the features to a certain extent. An artificial neural network (ANN) was established using the 10 independent factors of clinically significant prostate cancer noted above (24). The network ANN, logistic regression (LR) (25), support vector machine (SVM) (26), K-nearest neighbor (KNN) (27), decision tree (DT) (28), random forest (RF) (29), and six common machine learning models were analyzed. The results showed that the sensitivity of the ANN model to significant prostate cancer was the highest, up to 0.80, and the sensitivity of SVM was the lowest, at 0.2. The specificity of the six models for CS-PCa was 0.95 in the SVM model and 0.81 in the Decision Tree model. The F1 scores of the six machine learning models were all high, with the highest being the ANN model (0.897) and the lowest being the Decision Tree model (0.847). In the Youden index, the SVM model had the lowest score (0.155), while the ANN model had the highest score (0.686). In the ROC curve, the AUC was 0.855 in the ANN model, slightly higher than the AUC value of 0.845 in the SVM model. Based on the analysis of six evaluation indicators, the ANN model was better than the other five models for predicting CS-PCa, and it had the best prediction effect. In consistency verification of the machine learning model, the Kappa coefficient of ANN was 0.616 (0.61–0.80 high consistency), and the Kappa coefficient of the other five models was lower than 0.60 (0.41–0.60 medium consistency). The effective evaluation of the machine learning models is shown in **Table 2**, and the ROC curve is shown in **Figure 5**.

**Table 2:** Results for six machine learning models evaluated for the efficacy of clinically significant prostate cancer test data.

ML Models	Sensitivity	Specificity	PPV	NPV	Youden	F1	Kappa
ANN	0.8 (0.6996, 0.9004)	0.886 (0.8062, 0.9658)	0.706 (0.5917,0.8203)	0.929 (0.8645,0.9935)	0.686 (0.5695,0.8025)	0.8966 (0.8202, 0.973)	0.6164 (0.4944, 0.7384)
LR	0.5333 (0.4081, 0.6585)	0.886 (0.8062, 0.9658)	0.615 (0.4929, 0.7371)	0.848 (0.7579, 0.9381)	0.419 (0.2952, 0.5428)	0.8667 (0.7814, 0.952)	0.439 (0.3145, 0.5635)
SVM	0.2 (0.0996, 0.3004)	0.955 (0.903, 1.007)	0.6 (0.4771, 0.7229	0.778 (0.6737. 0.8823)	0.155 (0.0642, 0.2458)	0.8571 (0.7693, 0.9449)	0.439 (0.3145, 0.5635)
RF	0.6 (0.4771, 0.7229)	0.886 (0.8062, 0.9658)	0.643 (0.5228, 0.7632)	0.867 (0.7818, 0.9522)	0.486 (0.3606, 0.6114)	0.8764 (0.7938, 0.959)	0.4973 (0.3718, 0.6228)
DT	0.6667 (0.5848, 0.785)	0.818 (0.7212, 0.9148)	0.556 (0.4313, 0.6807)	0.878 (0.7959, 0.9601)	0.495 (0.3695, 0.6205)	0.8471 (0.7568, 0.9374)	0.4549 (0.3299, 0.5799)
KNN	0.5333 (0.4081, 0.6585)	0.864 (0.778, 0.95)	0.571 (0.4468, 0.6952)	0.844 (0.7529, 0.9351)	0.397 (0.2742, 0.5198)	0.8539 (0.7653, 0.9425)	0.4059 (0.2827, 0.5291)

## 4 Discussion

Depending on the applied prostate-specific antigen (PSA) cutoff as a trigger for a prostate biopsy, PSA can be a highly sensitive marker for prostate cancer (PCa) screening. However, up to 20% of clinically significant PCa (CS-PCa) can be missed on the first systematic TRUS-guided prostate biopsy in men with low PSA values (30–32). The relevant indicators of PSA should exhibit a certain value for predicting CS-PCa. In this study, unary logistic regression analysis was performed to determine the positive correlation of tPSA, fPSA, f/tPSA, PSAD, and other factors with CS-PCa (P< 0.001), while the fPSA/tPSA ratio was negatively correlated with CS-PCa (P< 0.001). The results obtained agree with those of Zhou (33). PSAD is an indicator that reflects the PSA per unit volume of the prostate, so it shows higher sensitivity and specificity than PSA (34). Studies have highlighted that PSAD is an important factor for predicting the postoperative Gleason score (GS), especially when GS< 7. The combined age of PSAD is of great value for the increase in GS after an operation (35). Hansen found that a PSAD cutoff value > 0.15 ng/mL/mL significantly improved the positive predictive value (PPV) (33%) for csPCa when performing targeted biopsies for PI-RADS 3 lesions (36). Venderink reported that using a PSAD cutoff value ≥ 0.15 ng/mL/mL for patients with PI-RADS 3 lesions resulted in 42% of the patients avoiding targeted biopsies, and 6% of cases of csPCa being missed (37). In this study, the age of the CS-PCa group was higher than that of the Non-CS-PCa group (75 vs. 73 years, P = 0.017), and the PSAD of the CS-PCa group was significantly higher than that of the Non-CS-PCa group (0.55 vs. 0.17, P< 0.001). Some studies have shown that prostate health index (PHI) is the best PSA-derived biomarker to predict CS-PCa in men with PI-RADS 3 lesions in a cognitive MRI-TRUS fusion targeted biopsy. PHI exhibits the highest prediction performance for CS-PCa with an AUC of 0.884, which is higher than that of PSAD. Targeted biopsies show more CS-PCa and less Non-CS-PCa than systematic biopsies (38).

Prostate MRI has developed into an important tool for the management of PCa. Prostate MRI is recommended as the first-line screening method for patients with a clinical suspicion of prostate cancer (39). The Prostate Imaging-Reporting and Data System (PI-RADS) represents a comprehensive set of guidelines, standardized observations, and lexicon, which aim to stratify the probability of clinically significant prostate cancer (csPCa) for MRI (40). In this study, the detection rates of CS-PCa and Non-CS-PCa were statistically analyzed by multimodal ultrasound scoring systems such as the 2D score, electrograph score, and CEUS score. The results showed that the differences were statistically significant (P< 0.001). The tumors of the Non-CS-PCa group showed high differentiation, low invasiveness, and relatively unobvious malignant signs in two-dimensional ultrasound images, whereas the tumors of the CS-PCa group showed low differentiation, high invasiveness, and proliferation of a large number of tumor cells, which may show an obvious space-occupying effect to form nodules, and may also involve the prostatic capsule and adjacent organs. PCa with different GS also exhibited different sonogram features. This resulted in a difference in the multimodal ultrasound scores of the Non-CS-PCa and CS-PCa groups. At present, the most widely used method for the clinical diagnosis of PCa is an ultrasound-guided transmittal prostate biopsy. However, the main limitations of this method are a large number of false negatives, the frequent failure to detect CS-PCa, and excessive puncture, which can cause misdiagnosis, missed diagnosis, and an increase in the incidence of complications after the examination. Grey used mpMRI to predict prostate biopsy results, and csPCa (ROC) curve analysis showed that the area under the curve (AUC) was 0.89, while the negative predictive value (NPV) of the PI-RADS Score 2 was 0.9814. However, the positive predictive value (PPV) of csPCa was 0.49, and a small number of patients with PI-RADS scores of 2 had CSPCa (41). The PROMIS trial was designed to evaluate the utility of mpMRI as a triage test in biopsy-naive patients to avoid unnecessary TRUS biopsy. The diagnostic accuracy of mpMRI and TRUS-guided biopsy was compared using transparency template prostate mapping as the reference standard. Ahmed reported that using mpMRI as a triage test resulted in avoiding 27% of primary biopsies, reducing the detection rate of clinically insignificant cancer by 5%, and improving the detection rate of csPCa by 18% (42). Wagner reported that the AUC of the model for PCa and csPCa prediction in patients with negative prostate MRI was 0.80 and 0.87, respectively, which showed that the number of biopsies in patients with negative MRI examination could be significantly reduced without a significant loss of CS-PCa detection (43).

Endemics can quantitatively analyze imaging data to noninvasively evaluate the biological behavior of tumors and has been widely used in the diagnosis, invasiveness assessment, and clinical decision-making of PCa (44). Radiolysis methods have shown high predictive performance in the diagnosis of benign and malignant PCa based on GS scores (45). Machine learning is the core of artificial intelligence, which mainly studies how computers can build models from data and use the established models to make predictions on new inputs. At present, the machine-learning algorithms widely used in the medical field include ANN, SVM, KNN, DT, RF, and LR. Chen studied the multi-gene model constructed by machine learning to predict prostate cancer and concluded that the accuracy of the RF model to identify prostate cancer was 94% with AUC = 0.94. Wang conducted a machine-learning prediction study of prostate cancer, using transmittal ultrasound video clips, and found that the AUCs of the SVM model in the validation set and test set were 0.78 and 0.75, respectively, and the diagnostic efficiency of the SVM model was higher than that of MRA-based diagnosis (AUC was 0.78 vs. 0.65/0.75 and 0.75 vs. 0.65/0.72, respectively). Li found that the MRI-based SVM model had high diagnostic efficiency and stability (46). In this study, the accuracy of the SVM model for the diagnosis of clinically significant prostate cancer was 95%, and the AUC was 0.845, which is similar to the effect in Li’s study. The MRI radiomics model based on T2WI and ADC constructed by Li can improve the diagnosis of csPCa, and its efficacy in the validation set (AUC = 0.98) (47) is higher than that of the ANN model in this study (AUC = 0.855). This outcome may be due to the inclusion of ADC and clinical risk factors in Li’s study. Chen also constructed a radiomics model and found that the radiomics model showed higher predictive performance than a PI-RADS v2 evaluation in the identification and invasiveness assessment of PCa. In the identification of PCa, the AUC of the model in the validation set was 0.999, and the AUC of the invasiveness assessment was 0.93 (48), which were higher than those in this study. In addition, Nketiah combined the texture features extracted from T2WI, quantitative ADC values, and DCE pharmacokinetics parameters to predict the GS grade of PCa, and its prediction performance (AUC = 0.91) was higher than that of the ANN model in this study (AUC = 0.855). This outcome may be due to the small number of patients included (23 cases) and the lack of an independent validation set (49).

In this study, six machine-learning models for the detection of prostate cancer were established based on the significant prostate cancer risk factors identified by single logistic analysis. The prostate cancer machine-learning model was comprehensively evaluated based on the model evaluation indexes. The results showed that the machine-learning models could effectively detect CS-PCa categories, and the ANN model was the best (sensitivity was 0.80%, accuracy was 88.6%, and AUC was 0.855). Porter studied six widely used prediction models and found that ANN was better than the other models in predicting the outcome of a prostate biopsy. Furthermore, when more input variables are used before the biopsy, many unnecessary biopsies can be avoided. This is consistent with the results obtained in this study (50). Nevertheless, the current study still has some shortcomings. First, this study was conducted at a single institution, so the number of cases was small. Subsequently, the sample was expanded and combined with multi-center clinical studies to verify the results. Second, this study only included prostate cancer cases and only evaluated the aggressiveness of prostate cancer. In the future, a study on the differential prediction of benign and malignant prostate cancer may be conducted. Third, the pathology of the prostatic biopsy was used as the control rather than the pathology of a radical prostatectomy, which may lead to bias (51). The final multimodal ultrasound test data are subjective to a certain extent. In particular, ultrasonic elasticity imaging relies on manual pressure, and the pressure intensity and frequency of different physicians differ, which may cause certain deviations in the elasticity scores.

## 5 Conclusion

In our study, we innovatively established machine learning to predict CS-PCa using PSA-related risk factors. The predictive model based on machine learning has superior diagnostic efficiency in CS-PCa. In the ANN model, the sensitivity, PPV and NPV of the model are higher than the other five machine learning models. So machine learning models based on psa related risk factors can help radiologists improve their diagnosis. In future work, we intend to combine various machine learning models and deep learning models in the application of CS-PCa ultrasound diagnosis to improve the diagnosis of significant prostate cancer. In conclusion, establishing a machine learning model using CS-PCa risk factor groups can be an important method for detecting PCa. Multimodal ultrasound combined with PSA-related indicators has high clinical value in the diagnosis of CS-PCa.

## Data availability statement

The original contributions presented in the study are included in the article/**Supplementary Materials**, further inquiries can be directed to the corresponding author/s.

## Ethics statement

The studies involving human participants were reviewed and approved by the Ethics Committee of Dongyang People’s Hospital. Written informed consent for participation was not required for this study in accordance with the national legislation and the institutional requirements. Written informed consent was not obtained from the individual(s) for the publication of any potentially identifiable images or data included in this article.

## Author contributions

Conceptualization, MZ, YL, KW and ZW; methodology, MZ, YL, KW and ZW; validation, MZ and YL; formal analysis, JY and LC; investigation, ZW and YD; resources, MZ and ZW; data curation, KW, JT, ZH and YJ; writing—original draft preparation, MZ and YL; writing—review and editing, YD and XS; visualization, MZ and YL; supervision, ZW and LC; project administration, MZ and ZW; funding acquisition, ZW and LC. All authors have read and agreed to the published version of the manuscript.
